# 人肺腺癌细胞系Ch-Huang-1的建立和生物学特性研究

**DOI:** 10.3779/j.issn.1009-3419.2012.05.03

**Published:** 2012-05-20

**Authors:** 白翎 李, 铿 钟, 磊 金, 扬 袁, 德军 龚, 晓红 刘, 盛东 黄

**Affiliations:** 200433 上海，第二军医大学附属长海医院胸心外科 Department of Cardiothoracic Surgery, Changhai Hospital, the Second Military Medical University, Shanghai 200433, China

**Keywords:** 肺肿瘤, 细胞系, 生物学特性, Lung neoplasms, Cell line, Biological characteristics

## Abstract

**背景与目的:**

肺癌是严重威胁人类生存和发展的恶性疾病之一，本研究旨在建立人肺腺癌细胞系，探讨其生物学特性，为进一步明确肺癌的发生机理，提供一个技术平台。

**方法:**

取人肺腺癌新鲜手术肿瘤标本，经组织块体外培养并克隆建系，命名为Ch-Huang-1。采用光镜、细胞生长曲线、染色体核型分析、克隆形成率及免疫缺陷小鼠成瘤实验对细胞系生物学特性进行分析。

**结果:**

细胞系及移植瘤标本证实具有腺癌恶性细胞特征。生长曲线呈S型，细胞群体倍增时间为36 h。分裂指数曲线为倒V字型，培养第30小时处于分裂期的细胞最多。克隆形成率为0.803%±0.078%。观察到肿瘤细胞染色体数目为亚三倍体，有明显的染色体异常。裸鼠皮下异种移植形成移植瘤。

**结论:**

根据细胞系特征显示该细胞系是一新建的肺腺癌细胞系。

## Abstract (en)

**Background and objective:**

Lung cancer is a major worldwide health problem. The aim of this study is to establish a novel Chinese human lung adenocarcinoma cell line and examine its biological characteristics.

**Methods:**

Lung adenocarcinoma specimens were freshly resected during surgery. The tissues were incubated *in vitro* and the cell line was named Ch-Huang-1. The biological characteristics of the cells were investigated by light microscopy, chromosome analysis, and transplantation experiment.

**Results:**

Light microscopy revealed that cells from the primary tumor, Ch-Huang-1 cell line, and transplanted tumor possessed the characteristics of a malignant glandular epithelial tumor. The cell growth curve, doubling time, and mitotic index were also observed *in vitro*. Nuclear chromosome analysis revealed that the tumor was a subtriploid with a mode of 35-44 per cell. Tumor nodes were observed under the skin of nude mice by heterogenic transplantation.

**Conclusion:**

The characteristics of the established cell line suggest that it is a newly established human adenocarcinoma cell line.

肺癌已成为当今世界发病率和死亡率增长最快且严重危害人类健康和生命的恶性肿瘤；而肺腺癌是肺癌中最常见的组织类型之一，且发病率逐年上升^[1]^。因此，对肺腺癌的研究显得尤为重要。建立肺腺癌细胞系对于研究肺癌癌变、侵袭转移、多药耐药的分子机制、生物学特征以及开发抗肺癌新药等均有十分重要的理论和临床意义。由于肺是一个开放性器官，其肿瘤组织易受细菌、霉菌的污染，较难培养^[2]^。我们利用1例原发性肺腺癌的肿瘤手术标本，采用原代培养方法建立了一个新的细胞系Ch-Huang-1，为肺腺癌研究工作提供新的实验材料。

## 1 材料与方法

### 1.1 标本来源

标本取自长海医院胸心外科46岁女性原发性肺腺癌Ⅲb期患者。病理诊断：右肺下叶低分化腺癌。支气管断端“-”，肺门淋巴结、第8组淋巴结癌转移“+”。

### 1.2 培养方法

将肿瘤标本浸入含双抗的RPMI-1640培养液中，送胸心外科实验室。在无菌条件下，用含有10%胎牛血清和青霉素及链霉素（各100 mg/mL）的PBS冲洗，剔除血管、包膜和坏死组织后剪成约1 mm^3^的组织块，充分洗涤。在无菌条件下，将剪好的组织块贴于含少量培养液的25 mL培养瓶壁上，每瓶10块，置于37 ℃、CO_2_孵箱。4 h后接触培养液，使组织块浸于培养液中^[3]^。4 d后半量换液。20 d后出现细胞克隆，将克隆挑出，胰酶消化传代1次。细胞长至80%左右传代。早期细胞形态以贴壁细胞为主，多为大小不等的多边形，核大，可见分裂相。分裂后细胞仍呈贴壁状，细胞异质性明显。

### 1.3 生长曲线

取对数生长期的第5代细胞经胰酶消化后，稀释成细胞悬液进行计数，并调整浓度为每毫升细胞悬液含100, 000个细胞，然后0.1 mL/孔（100个细胞）分别加入到24孔板的相应孔中，并用完全培养基补足体积至0.5 mL。每天取出3个孔的细胞进行计数，连续9 d进行细胞计数。以培养时间为横轴，细胞数为纵轴，绘制生长曲线。

### 1.4 分裂指数

取第5代细胞悬液接种于事先放置有小盖玻片的6孔板内，接种要求同生长曲线。每24 h取出一个玻片，按常规方法95%酒精固定，Giemsa染色。选择细胞密度适中的区域观察分裂细胞，进行细胞计数。每个时间组的玻片各取细胞数多、中、少各一区，共1, 000个细胞。

### 1.5 克隆形成率

接种分散均匀的第5代细胞于6孔板中，每孔1, 000个细胞，静止培养2周-3周。当培养板中出现肉眼可见的克隆时，终止培养。甲醇固定加适量Giemsa染色，计数克隆数。

### 1.6 组织起源鉴定

细胞悬液种到无菌载玻片，含10%胎牛血清的DMEM培养至细胞完全伸展、贴壁，PBS洗，滴加SPC抗体，37 ℃、1 h，PBS洗，滴加FITC标记的二抗，37 ℃避光30 min，荧光显微镜下观察。

### 1.7 染色体分析

在第5代细胞的对数生长期加秋水仙素作用2 h，经0.075 mol/L KCl低渗、甲醇-冰醋酸（3:1）固定处理，冰湿片滴片，室温老化后用胰酶处理，Giemsa染色显带分析。

### 1.8 免疫缺陷小鼠成瘤实验

在4周-6周龄小鼠背部皮下注射2×10^6^个/0.2 mL收获呈指数生长的培养细胞，1代-4代细胞60 d后仍不致瘤，传至第5代才致瘤。1周时可触摸到肿块生长，观察瘤结节形成情况。

## 2 结果

### 2.1 形态现察

活细胞形态采用组织块贴壁法原代培养细胞（图 1A），联合采用机械刮除、反复贴壁等方法去除成纤维细胞，建立可传代细胞系；倒置显微镜下细胞大小不等，癌细胞呈团块状分布，细胞体积小，呈圆形，细胞铺满瓶底后有重叠现象。目前，所建立的肺腺癌可传代细胞系已传至第70代，细胞形态和生长速度不变。冻存后复苏良好，可以认为基本建系。从第2代传代后细胞多呈多角形。少数为梭形或圆形（图 1B）。

**1:**
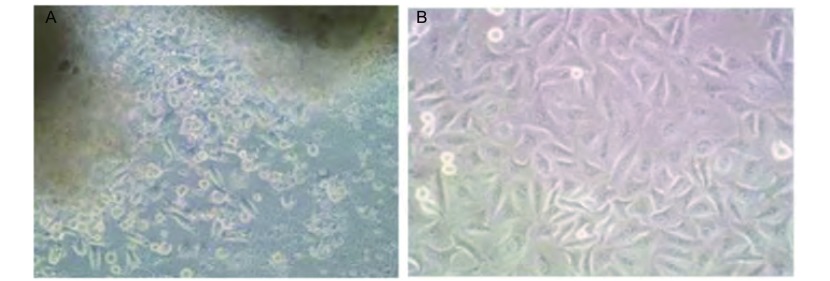
倒置显微镜光镜观察：细胞多呈多角形，少数为梭形或圆形（×200）。A：原代培养细胞；B：传代细胞。 Most cells were polygonal and a few were spindle or round (×200). A: primary cultured cells; B: passage cells.

### 2.2 生长曲线

生长曲线呈S型；在生长曲线的对数生长期取细胞数成倍生长的两个点作垂直线测定时间，细胞群体倍增时间为36 h（图 2）。

**2:**
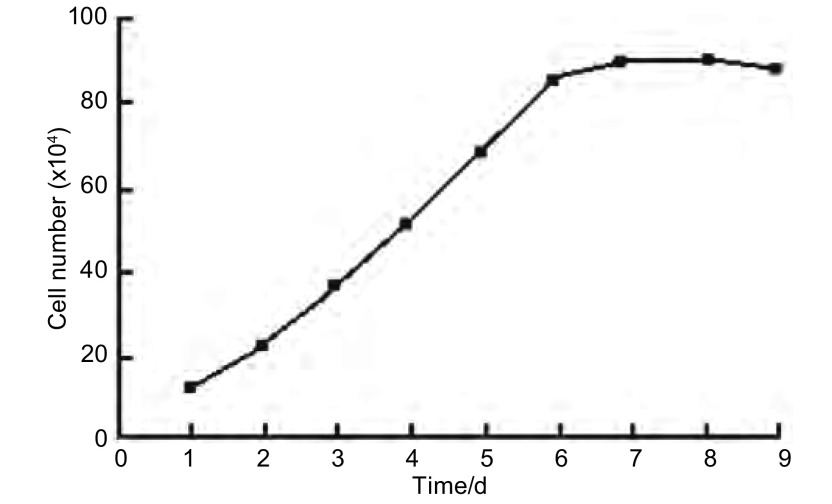
细胞生长曲线图 Growth curve of cells line Ch-Huang-1

### 2.3 分裂指数

分裂指数=分裂相细胞数平均值/总细胞数平均值（图 3），分裂指数曲线为倒V字型，培养第30小时处于分裂期的细胞最多。

**3:**
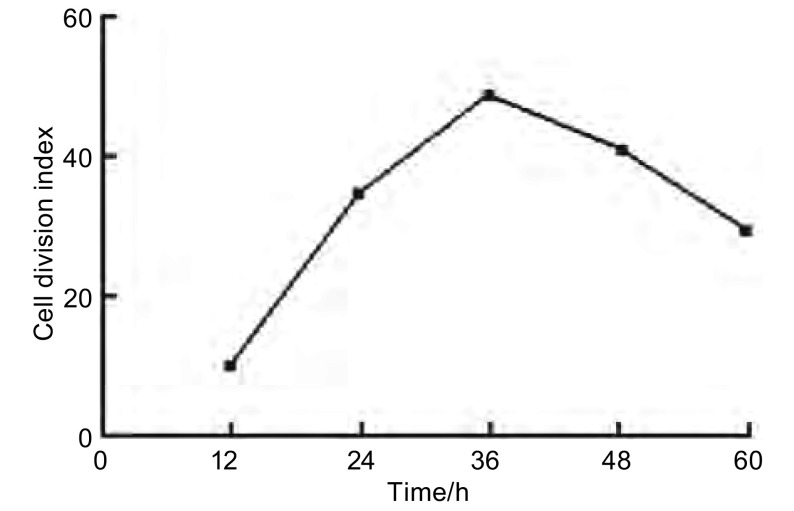
细胞分裂指数分析 Analysis of cells division index

### 2.4 克隆形成率

计算出克隆形成率：克隆形成率=克隆数/接种细胞数×100%，本研究克隆形成率为0.803%±0.078%。

### 2.5 组织起源鉴定

荧光显微镜下可以观察到90%的培养细胞表达Ⅱ型肺泡（alveolar type Ⅱ, ATⅡ）上皮细胞标志蛋白：疏水性表面活性蛋白C（surfactant protein C, SPC）（图 4），提示其起源于ATII细胞。

**4:**
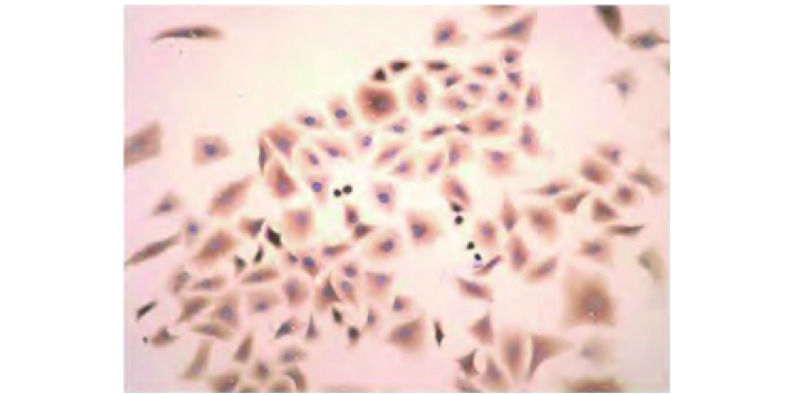
细胞组织起源鉴定（DAB，×200） Identification of the cellular tissue origin (DAB, ×200)

### 2.6 染色体分析

在显微镜下选择分散良好且比较完整的第12代中期分裂象进行观察，计数100个分裂象的染色体数目。观察到各细胞内染色体数目分布在35-44之间。染色体数目为亚三倍体，有明显的染色体异常（图 5）。

**5:**
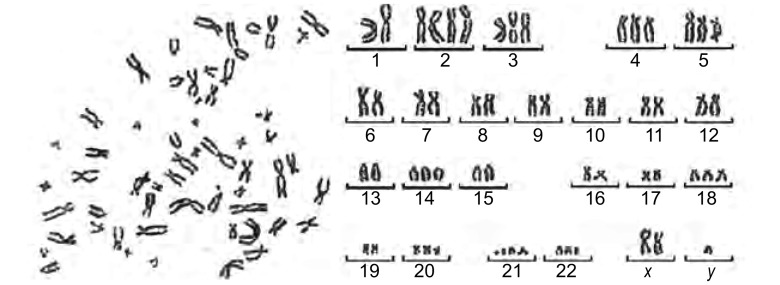
染色体核型分析 Chromosomal instability in Ch-Huang-1

### 2.7 免疫缺陷小鼠成瘤实验

裸鼠皮下种植1代-4代细胞均不致瘤，传至第5代后100%致瘤。4周瘤块长至直径约0.8 cm-1.0 cm左右时，用断颈法将鼠处死（图 6）。常规HE染色观察细胞生长旺盛，细胞核不均匀增大，异型性明显，其组织学形态与原发瘤相似。目前传代至第70代，致瘤效果未有衰退。

**6:**
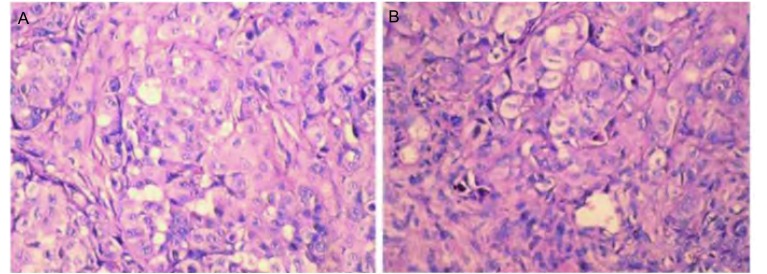
移植瘤癌细胞生长旺盛（HE，×40）。A：原代培养用组织块；B：移植瘤细胞。 Vigorous growth of transplanted tumor cells (HE, ×40). A: primary tissue culture cells; B: transplantation tumor cells.

## 3 讨论

应用体外建立的肿瘤细胞系进行研究，对揭示肿瘤的发病机制、寻找诊断肿瘤的有用指标和探索实验性的治疗方案都能提供十分有价值的参考资料。然而，成功建立一个原发肿瘤的永生化细胞系是很困难的。而作为一株合格的细胞系，应当符合以下标准^[4-6]^：在体外培养半年以上，生长稳定，能连续传代；细胞生长特性明确；有遗传学分析；肿瘤标记物测定；异种成瘤性测定；无污染。我们利用术中取得的肺腺癌标本，通过完整组织块原代培养、皮下移植瘤模型构建、皮下移植瘤原代培养等一系列方法建立肿瘤细胞系。本实验应用组织块法获得肺腺癌原代细胞，为建立细胞系奠定了基础。Ch-Huang-1细胞系至今在体外培养已经超过1年，且生长稳定，能够连续传代。Ch-Huang-1细胞系在体外培养过程中，接触性抑制消失，重叠性生长，反映了肿瘤细胞恶性增殖的特点^[7]^。

本细胞系所描绘的生长曲线细胞呈“S”型，符合无限细胞系的特点。软琼脂克隆形成实验结果显示，Ch-Huang-1细胞具有非贴壁依赖性生长特性，能够进行自主性增殖，表现出恶性肿瘤细胞的重要特征^[8]^。通过细胞核型分析显示：来源于人类的非整倍体细胞、多倍体细胞的出现代表着基因拷贝量的大量增加、核分裂加速、细胞增殖失控、核浆分裂失衡等细胞恶性增殖特性，异常染色体及端着丝粒等染色体结构异常可能与细胞锚着独立性生长能力、生长增殖优势及成瘤性等恶性细胞特性有明显关联^[9-11]^。本研究对细胞核型进行分析后显示该细胞系存在染色体数量和结构的异常。我们观察到各细胞内染色体数目分布在35-44之间。染色体数目为亚三倍体，有明显的染色体异常。这为以后从遗传学角度寻找肺腺癌形成机制提供了实验资料。

本研究显示我们所建Ch-Huang-1细胞系具有典型的恶性细胞形态特点；免疫缺陷小鼠经接种细胞后产生移植瘤，其组织病理学特征与原发肿瘤特征一致。经长期传代培养其形态学、细胞动力学及染色体众数等指标无明显改变，表明该细胞系生长稳定，符合一般体外培养细胞系标准。

综上所述，人肺腺癌细胞系Ch-Huang-1的建立可为深入研究肺腺癌的发病机制、生物学行为提供细胞学实验模型，并为进一步开展分子生物学、免疫学及临床诊断、治疗等研究提供有利条件。
